# Secular trends in cholesterol lipoproteins and triglycerides and prevalence of dyslipidemias in an urban Indian population

**DOI:** 10.1186/1476-511X-7-40

**Published:** 2008-10-24

**Authors:** Rajeev Gupta, Soneil Guptha, Aachu Agrawal, Vijay Kaul, Kiran Gaur, Vijay P Gupta

**Affiliations:** 1 Department of Medicine, Fortis Escorts Hospital, Jaipur 302017, India; 2 Departments of Medicine and Pathology, Monilek Hospital and Research Centre, Jaipur 302004, India; 3 Regional Headquarters, MSD Technology Singapore Pte Ltd, 188778, Singapore; 4 Department of Home Science, University of Rajasthan, Jaipur 302004, India; 5 Department of Statistics, University of Rajasthan, Jaipur 302004, India

## Abstract

**Background:**

Coronary heart disease is increasing in urban Indian subjects and lipid abnormalities are important risk factors. To determine secular trends in prevalence of various lipid abnormalities we performed studies in an urban Indian population.

**Methods:**

Successive epidemiological Jaipur Heart Watch (JHW) studies were performed in Western India in urban locations. The studies evaluated adults ≥ 20 years for multiple coronary risk factors using standardized methodology (JHW-1, 1993–94, n = 2212; JHW-2, 1999–2001, n = 1123; JHW-3, 2002–03, n = 458, and JHW-4 2004–2005, n = 1127). For the present analyses data of subjects 20–59 years (n = 4136, men 2341, women 1795) have been included. In successive studies, fasting measurements for cholesterol lipoproteins (total cholesterol, LDL cholesterol, HDL cholesterol) and triglycerides were performed in 193, 454, 179 and 252 men (n = 1078) and 83, 472, 195, 248 women (n = 998) respectively (total 2076). Age-group specific levels of various cholesterol lipoproteins, triglycerides and their ratios were determined. Prevalence of various dyslipidemias (total cholesterol ≥ 200 mg/dl, LDL cholesterol ≥ 130 mg/dl, non-HDL cholesterol ≥ 160 mg/dl, triglycerides ≥ 150 mg/dl, low HDL cholesterol <40 mg/dl, high cholesterol remnants ≥ 25 mg/dl, and high total:HDL cholesterol ratio ≥ 5.0, and ≥ 4.0 were also determined. Significance of secular trends in prevalence of dyslipidemias was determined using linear-curve estimation regression. Association of changing trends in prevalence of dyslipidemias with trends in educational status, obesity and truncal obesity (high waist:hip ratio) were determined using two-line regression analysis.

**Results:**

Mean levels of various lipoproteins increased sharply from JHW-1 to JHW-2 and then gradually in JHW-3 and JHW-4. Age-adjusted mean values (mg/dl) in JHW-1, JHW-2, JHW-3 and JHW-4 studies respectively showed a significant increase in total cholesterol (174.9 ± 45, 196.0 ± 42, 187.5 ± 38, 193.5 ± 39, 2-stage least-squares regression R = 0.11, p < 0.001), LDL cholesterol (106.2 ± 40, 127.6 ± 39, 122.6 ± 44, 119.2 ± 31, R = 0.11, p < 0.001), non-HDL cholesterol (131.3 ± 43, 156.4 ± 43, 150.1 ± 41, 150.9 ± 32, R = 0.12, p < 0.001), remnant cholesterol (25.1 ± 11, 28.9 ± 14, 26.0 ± 11, 31.7 ± 14, R = 0.06, p = 0.001), total:HDL cholesterol ratio (4.26 ± 1.3, 5.18 ± 1.7, 5.21 ± 1.7, 4.69 ± 1.2, R = 0.10, p < 0.001) and triglycerides (125.6 ± 53, 144.5 ± 71, 130.1 ± 57, 158.7 ± 72, R = 0.06, p = 0.001) and decrease in HDL cholesterol (43.6 ± 14, 39.7 ± 8, 37.3 ± 6, 42.5 ± 6, R = 0.04, p = 0.027). Trends in age-adjusted prevalence (%) of dyslipidemias in JHW-1, JHW-2, JHW-3 and JHW-4 studies respectively showed insignificant changes in high total cholesterol (26.3, 35.1, 25.6, 26.0, linear curve-estimation coefficient multiple R = 0.034), high LDL cholesterol ≥ 130 mg/dl (24.2, 36.2, 31.0, 22.2, R = 0.062), and high low HDL cholesterol < 40 mg/dl (46.2, 53.3, 55.4, 33.7, R = 0.136). Increase was observed in prevalence of high non-HDL cholesterol (23.0, 33.5, 27.4, 26.6, R = 0.026), high remnant cholesterol (40.1, 40.3, 30.1, 60.6, R = 0.143), high total:HDL cholesterol ratio ≥ 5.0 (22.2, 47.6, 53.2, 26.3, R = 0.031) and ≥ 4.0 (58.6, 72.5, 70.1, 62.0, R = 0.006), and high triglycerides (25.7, 28.2, 17.5, 34.2, R = 0.047). Greater correlation of increasing non-HDL cholesterol, remnant cholesterol, triglycerides and total:HDL cholesterol ratio was observed with increasing truncal obesity than generalized obesity (two-line regression analysis p < 0.05). Greater educational level, as marker of socioeconomic status, correlated significantly with increasing obesity (r^2 ^men 0.98, women 0.99), and truncal obesity (r^2 ^men 0.71, women 0.90).

**Conclusion:**

In an urban Indian population, trends reveal increase in mean total-, non-HDL-, remnant-, and total:HDL cholesterol, and triglycerides and decline in HDL cholesterol levels. Prevalence of subjects with high total cholesterol did not change significantly while those with high non-HDL cholesterol, cholesterol remnants, triglycerides and total-HDL cholesterol ratio increased. Increasing dyslipidemias correlate significantly with increasing truncal obesity and obesity.

## Introduction

Cardiovascular diseases, especially coronary heart disease, are important public health problems in India and many developing countries [1,2]. There is evidence that the diseases are increasing in these countries in contrast to developed nations of Europe and North America where the incidence has decreased [3,4]. Societal changes as well as individual lifestyle factors are important in driving this cardiovascular epidemic [5]. These changes influence the proximate determinants of atherosclerosis which include smoking and tobacco use, high total and low density lipoprotein (LDL) cholesterol, low high density lipoprotein (HDL) cholesterol, high blood pressure, diabetes and the metabolic syndrome. Trends of these risk factors have been well studied in developed countries and show significant correlation with rise and fall of the coronary heart disease epidemic [5].

There have been only a few studies that have examined trends in cardiovascular risk factors in middle and low income countries [6]. In Seven Countries Study multiple cross sectional surveys were conducted among men aged 40–59 years in Yugoslavia, Italy, Greece, Holland, Finland, Japan and USA [7]. These studies reported that while major coronary risk factors initially stabilized and later declined in many of these countries, in middle income countries such as Yugoslavia the risk factors increased. The WHO-MONICA study reported that population risk factors increased in the Chinese while they declined in North American and Western European cohorts [8,9]. Increasing trends in coronary risk factors has also been reported from many middle income Latin American countries [6]. In Asia, increasing trends in lipids and in prevalence of dyslipidemias (high LDL cholesterol and low HDL cholesterol) has been reported in urban populations of Beijing [10], rural China [11] and South Korea [12].

To our knowledge no single study that systematically evaluated trends in major cardiovascular risk factors in India exists although reviews have reported increasing prevalence of hypertension [13], diabetes [14], and hypercholesterolemia [2], and declining smoking rates among the educated Indians [15]. All these evaluations suffer from multiple biases inherent in compiling studies from different sources and different methodologies [16]. We performed multiple coronary heart disease risk factor epidemiological studies in urban populations in western Indian state of Rajasthan to determine their lifestyle and other determinants [17-20]. Here we report trends in levels of various lipoproteins (total, LDL, HDL and non-HDL cholesterol, triglycerides) and total-HDL cholesterol ratio and prevalence of dyslipidemias using current definitions.

## Methods

A series of cross sectional epidemiological studies using similar tools in the Indian state of Rajasthan over years 1992–2005 were performed to determine cardiovascular risk factors in urban populations [17-20]. All the studies were approved by the institutional ethics committee and supported financially by different organizations. The studies have been performed in Jaipur, the capital city of Rajasthan state in western India with population in year 2001 of 2.34 million. The first study – Jaipur Heart Watch (JHW)-1 [17], was conducted in years 1993–1994 and randomly selected 1608 men and 1392 women were targeted using stratified cluster sampling on the Voters' lists in six locations in Jaipur city. 2212 subjects (1415 men 88.0%, 797 women 57.3%) were evaluated for various cardiovascular risk factors and attempt for fasting blood sample for cholesterol lipoproteins and triglycerides was in 15%. In the second urban study (JHW-2) [18] we targeted 960 men and 840 women in the same locations as in JHW-1 and could examine 550 men (57.3%) and 573 women (68.2%). In this study we targeted all the participants for the fasting blood sample. The third (JHW-3) [19] and fourth (JHW-4) [20] urban studies targeted at a smaller sample and were designed to gather information on risk factors in middle-class locations. Response rates are shown in Table 1.

**Table 1:** Demographic characteristics of various Jaipur Heart Watch studies

**Study**	**Years performed (Published)**	**Target sample size** **Age ≥ 20 years**		**Study subjects** **Age ≥ 20 years**		**Total subjects** **Age 20–59 years**		**Blood samples in enrollees** **Age 20–59 years**	
		**Men**	**Women**	**Men**	**Women**	**Men**	**Women**	**Men**	**Women**

**JHW-1**	1993–1995 (1995)	1608	1392	1415 (88.0)	797 (57.2)	1294	655	193 (14.9)	83 (12.7)
**JHW-2**	1999–2001 (2002)	960	840	532(55.4)	559(66.5)	454	472	454(100.0)	472 (100.0)
**JHW-3**	2001–2002 (2004)	320	280	226 (70.6)	232 (82.9)	179	195	179 (100.0)	195 (100.0)
**JHW-4**	2004–2005 (2007)	750	650	556 (74.1)	571 (87.8)	414	473	252 (60.9)	248 (52.4)
**Total**	N = 6790	3638	3152	2729 (75.0)	2159 (68.5)	2341	1795	1078 (46.0)	998 (55.6)

JHW Jaipur Heart Watch. Numbers in parentheses are percent response rates. Blood samples obtained for lipid profile estimation.

### Data collection

Methodological details have been previously reported [17]. Briefly, we collected information regarding demographic data, educational level, history of chronic illnesses such as coronary heart disease, hypertension, diabetes or high cholesterol levels, and smoking or tobacco intake. Brief questions were asked to evaluate physical activity and diet but the results were considered inadequate and not included in the analyses. Physical examination was performed to assess height, weight, waist and hip size and blood pressure. BMI was calculated as weight (kg) divided by squared height (m). Waist-to-hip ratio was calculated. Fasting glucose was determined at a central laboratory using glucose peroxidase method and external quality control. Total cholesterol was measured using cholesterol oxidase-phenol 4-aminophenazone peroxidase method and HDL cholesterol using an enzymatic method after precipitating non-HDL cholesterol with a managanese-heparin substrate. Triglycerides were measured using the glycerol phosphate oxidase-peroxidase enzymatic method. Quality control measures were followed for estimation of total cholesterol, high density lipoprotein (HDL) cholesterol and triglycerides while low density lipoprotein (LDL) cholesterol was estimated using the Friedewald formula [21].

### Diagnostic criteria

The diagnostic criteria for coronary risk factors have been advised by the American College of Cardiology clinical data standards [22]. Educational level was used as marker for socioeconomic status as reported in an earlier study [23]. More than 5 years of formal education (primary education or more) was taken as acceptable literacy level for analysis. Obesity or overweight was defined as body mass index of ≥ 25 kg/m^2 ^and truncal obesity was defined by waist:hip ratio of > 0.95 for men and > 0.85 for women [24]. Dyslipidemia was defined by the presence of high total cholesterol (≥ 200 mg/dl), high LDL cholesterol (≥ 130 mg/dl), low HDL cholesterol (< 40 mg/dl), high non-HDL cholesterol (≥ 160 mg/dl), high cholesterol remnants [very low density lipoprotein cholesterol = total – (HDL+LDL) cholesterol ≥ 25 mg/dl] or high triglycerides (≥ 150 mg/dl) according to National Cholesterol Education Program (NCEP) Adult Treatment Panel-3 (ATP-3) guidelines [25]. High total to HDL cholesterol was defined when ratio was either ≥ 5.0 or ≥ 4.0 as reported in an earlier study from India [26].

### Statistical analyses

The continuous variables are reported as mean ± 1 SD and ordinal variables in percent. Prevalence rates are reported in percent. Age-stratified prevalence rates and distribution of various risk factors have been reported for decadal intervals from 20 years 59 years. Age-adjustment of various prevalence rates was performed using direct method using the Jaipur urban population according to 2001 census. Correlation of age with lipid values was performed by simple correlation analysis and significance of age-adjusted trends in mean lipoprotein levels was evaluated by 2-stage least squares regression using SPSS 10.0 statistical package (SPSS Inc, Chicago). Significance of trends in prevalence rates was determined using linear curve-estimation regression analysis using the SPSS package. Regression coefficients are reported as multiple R values after age adjustment. Significance of graphical trends was determined by logarithmic regression analysis using the Microsoft Office Power Point (2002) program. Significance of two-line trends was determined by least squares regression analyses using GB-Stat for Windows^® ^software 7.0 (Dynamic Microsystems Inc, Silver Spring, MD USA) and reported as r^2 ^values. The r^2 ^values of more than 0.10 and p values less than 0.05 were considered significant.

## Results

The overall response rates in the study were 2747/3638 (76.5%) in men and 2173/3152 (68.5%) in women. Of the 4146 subjects aged 20–59 years (men 2341, women 1795), blood samples were obtained in 1078 (46.0%) men and 998 (55.6%) women (total = 2076, 50.1%). Response rates varied in different studies (Table 1).

**Figure 1:**
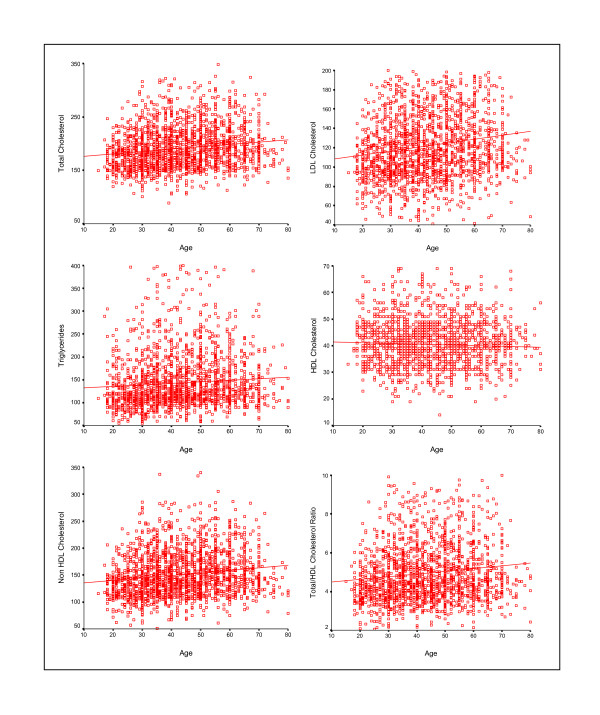
**Correlation of various cholesterol lipoproteins and triglycerides with age in combined data from JHW studies.** There is a significant increase in total cholesterol (r = 0.16), LDL cholesterol (r = 0.15), non-HDL cholesterol (r = 0.16), total: HDL cholesterol ratio (r = 0.13) and triglycerides (r = 0.07) with age (p < 0.001 for all). HDL cholesterol decreases slightly with age (r = -0.05, p = 0.02). Trends in men and women are almost similar and not reported separately.

**Figure 2:**
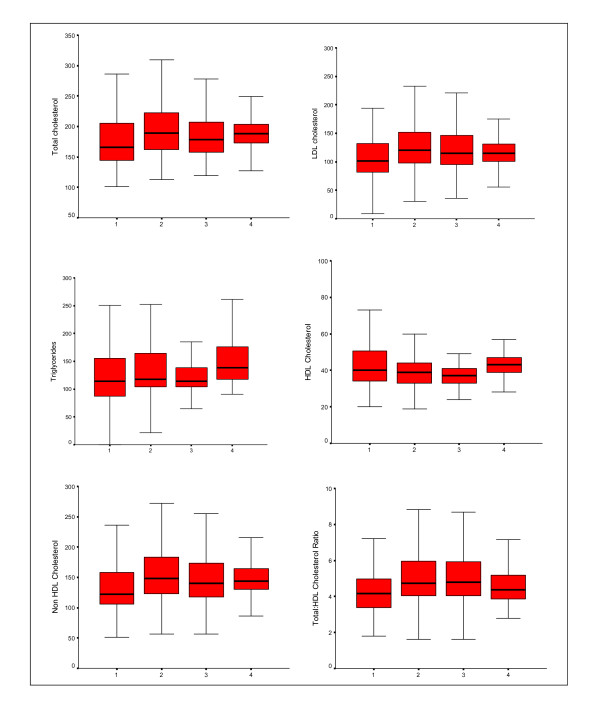
**Box-plot of mean and median values (mg/dl) and 95% confidence intervals of various cholesterol lipoproteins and triglycerides in various Jaipur Heart Watch Studies.** There is a significant increase in trends of age-adjusted levels of total cholesterol (2-stage least-squares regression R = 0.11, p < 0.001), LDL cholesterol (R = 0.11, p < 0.001), non-HDL cholesterol (R = 0.12, p < 0.001) and total: HDL cholesterol (R = 0.10, p < 0.001) and triglycerides (R = 0.06, p = 0.001) and decrease in HDL cholesterol (R = 0.04, p = 0.027).

Mean levels of various lipoproteins at different age-groups are shown in Table 2. There is age-associated escalation in total cholesterol, LDL cholesterol, non-HDL cholesterol, remnant cholesterol, total:HDL cholesterol ratio and triglycerides in men and women in all the cohorts. The levels of HDL cholesterol decline with age, the decline being similar in men and women. Correlation of various lipoproteins with age in combined data from JHW studies is shown in Figure 1. There is a significant increase in total cholesterol (r = 0.16), LDL cholesterol (r = 0.15), non-HDL cholesterol (r = 0.16), total:HDL cholesterol ratio (r = 0.13)and triglycerides (r = 0.07) with age (p < 0.001 for all). HDL cholesterol decreases slightly with age (r = -0.05, p = 0.02). Trends in men and women are almost similar and hence not reported separately. Comparison of levels across studies reveals increase in all lipid levels from JHW-1 to JHW-2 studies but are not significantly different in JHW-3 and JHW-4 studies. Age-adjusted trends in mean lipoprotein values (mg/dl) in JHW-1, JHW-2, JHW-3 and JHW-4 studies respectively show a significant increase in total cholesterol (174.9 ± 45, 196.0 ± 42, 187.5 ± 38, 193.5 ± 39, 2-stage least-squares regression R = 0.11, p < 0.001), LDL cholesterol (106.2 ± 40, 127.6 ± 39, 122.6 ± 44, 119.2 ± 31, R = 0.11, p < 0.001), non-HDL cholesterol (131.3 ± 43, 156.4 ± 43, 150.1 ± 41, 150.9 ± 32, R = 0.12, p < 0.001), remnant cholesterol (25.1 ± 11, 28.9 ± 14, 26.0 ± 11, 31.7 ± 14, R = 0.06, p = 0.001), total:HDL cholesterol (4.26 ± 1.3, 5.18 ± 1.7, 5.21 ± 1.7, 4.69 ± 1.2, R = 0.10, p < 0.001) and triglycerides (125.6 ± 53, 144.5 ± 71, 130.1 ± 57, 158.7 ± 72, R = 0.06, p = 0.001) and decrease in HDL cholesterol (43.6 ± 14, 39.7 ± 8, 37.3 ± 6, 42.5 ± 6, R = 0.04, p = 0.027) (Figure 2).

**Table 2:** Age-specific levels of cholesterol lipoproteins and triglycerides (mg/dl)

	**Men**				**Women**			
**Lipid parameters in mg/dl**	**JHW-1**	**JHW-2**	**JHW-3**	**JHW-4**	**JHW-1**	**JHW-2**	**JHW-3**	**JHW-4**

**Numbers**	**193**	**454**	**179**	**252**	**83**	**472**	**195**	**248**

Cholesterol								
20–29	163.9 ± 29	180.6 ± 35	167.1 ± 35	184.9 ± 29	174.0 ± 35	180.0 ± 33	171.4 ± 24	183.3 ± 19
30–39	182.1 ± 41	200.1 ± 47	189.6 ± 49	192.9 ± 25	174.9 ± 48	192.6 ± 41	193.1 ± 41	192.5 ± 48
40–49	183.6 ± 44	196.6 ± 43	194.7 ± 47	188.8 ± 28	161.9 ± 31	196.9 ± 37	194.7 ± 47	198.9 ± 49
170.7 ± 68	200.1 ± 41	200.3 ± 32	201.5 ± 31	190.8 ± 90	208.9 ± 45	200.3 ± 32	216.3 ± 53	
								
LDL cholesterol								
20–29	99.4 ± 27	113.7 ± 32	105.6 ± 27	110.5 ± 6	100.0 ± 30	114.3 ± 33	105.6 ± 27	112.4 ± 21
30–39	114.2 ± 38	127.1 ± 40	125.2 ± 46	116.5 ± 33	98.1 ± 37	126.2 ± 38	125.2 ± 46	119.3 ± 42
40–49	111.7 ± 44	128.1 ± 43	131.6 ± 49	110.1 ± 31	102.3 ± 42	130.3 ± 33	131.6 ± 49	123.0 ± 42
50–59	98.0 ± 52	129.5 ± 37	132.7 ± 34	127.4 ± 31	122.8 ± 71	137.1 ± 45	132.7 ± 34	138.3 ± 46
								
HDL cholesterol								
20–29	41.7 ± 12	40.5 ± 9	37.7 ± 8	43.0 ± 5	52.6 ± 22	41.6 ± 9	38.7 ± 5	44.7 ± 6
30–39	43.8 ± 10	39.1 ± 9	35.3 ± 8	41.3 ± 4	40.5 ± 11	38.9 ± 8	36.3 ± 6	42.6 ± 4
40–49	44.4 ± 11	38.3 ± 8	35.6 ± 5	43.0 ± 7	45.6 ± 15	38.5 ± 9	35.5 ± 5	42.7 ± 4
50–59	43.4 ± 20	40.1 ± 8	36.6 ± 5	39.6 ± 7	40.2 ± 15	41.2 ± 8	36.6 ± 5	42.6 ± 4
								
Non HDL cholesterol								
20–29	122.5 ± 30	140.1 ± 36.0	132.7 ± 24.8	141.9 ± 27.9	121.4 ± 34	138.4 ± 35.7	126.9 ± 31.4	138.6 ± 20.0
30–39	138.3 ± 41	161.0 ± 48.5	156.7 ± 43.1	151.6 ± 29.9	121.3 ± 35	153.7 ± 41.5	143.7 ± 35.8	142.8 ± 29.5
40–49	139.0 ± 43	159.9 ± 40.8	159.1 ± 49.5	145.7 ± 31.7	129.3 ± 45	158.2 ± 36.9	150.0 ± 35.6	145.1 ± 31.5
50–59	127.3 ± 56	150.7 ± 41.5	163.7 ± 34.8	161.8 ± 29.4	150.6 ± 79	167.7 ± 46.8	153.8 ± 44.8	164.2 ± 40.3
								
Total:HDL cholesterol ratio								
20–29	4.17 ± 1.0	4.67 ± 1.4	4.52 ± 0.9	4.38 ± 0.9	3.67 ± 1.3	4.53 ± 1.33	4.26 ± 1.3	4.17 ± 0.7
30–39	4.30 ± 1.1	5.40 ± 2.0	5.51 ± 1.7	4.87 ± 1.4	4.27 ± 1.4	5.19 ± 1.7	4.97 ± 1.5	4.45 ± 1.0
40–49	4.30 ± 1.2	5.37 ± 1.9	5.71 ± 2.2	4.54 ± 1.2	4.10 ± 1.4	5.28 ± 1.45	5.19 ± 1.4	4.55 ± 1.3
50–59	4.24 ± 1.4	5.18 ± 1.5	5.65 ± 1.5	5.27 ± 1.5	4.81 ± 1.4	5.29 ± 1.7	5.29 ± 1.7	5.03 ± 1.4
								
Triglycerides								
20–29	113.8 ± 56	131.8 ± 51	118.8 ± 42	157.3 ± 72	106.8 ± 41	120.1 ± 40	121.9 ± 38	130.8 ± 36
30–39	120.6 ± 48	169.5 ± 107	145.6 ± 118	175.6 ± 76	116.3 ± 46	137.3 ± 70	148.6 ± 117	153.1 ± 62
40–49	137.2 ± 50	150.8 ± 76	137.5 ± 57	178.0 ± 91	135.0 ± 42	140.9 ± 65	137.5 ± 57	161.2 ± 75
50–59	146.4 ± 75	152.2 ± 67	154.9 ± 63	172.2 ± 102	139.3 ± 57	153.2 ± 68	155.0 ± 63	176.5 ± 79
								
Remnant cholesterol								
l20–29	22.6 ± 11	26.4 ± 10	27.1 ± 19	31.5 ± 15	21.4 ± 8	24.0 ± 8	30.6 ± 51	26.1 ± 7
30–39	24.1 ± 10	33.9 ± 22	31.5 ± 26	35.1 ± 18	23.3 ± 9	27.5 ± 14	25.1 ± 18	29.9 ± 11
40–49	27.4 ± 10	30.2 ± 15	27.5 ± 11	35.6 ± 20	27.0 ± 8	27.9 ± 14	30.7 ± 45	30.4 ± 13
50–59	29.3 ± 15	30.4 ± 13	31.0 ± 13	34.3 ± 19	27.9 ± 11	30.6 ± 14	27.1 ± 12	31.4 ± 10

**Figure 3:**
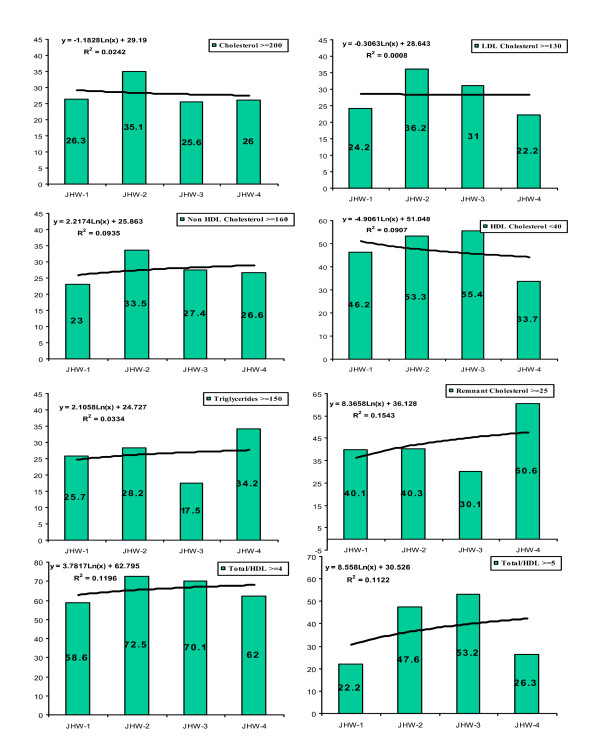
**Trends in age-adjusted prevalence of various dyslipidemias in various Jaipur Heart Watch (JHW) studies.** Graphic analysis shows that prevalence (%) of high total cholesterol ≥ 200 mg/dl (logarithmic r^2 ^= 0.026), high LDL cholesterol ≥ 130 mg/dl (r^2 ^= 0.001), low HDL cholesterol < 40 mg/dl (r^2 ^= 0.091) and triglycerides (r^2 ^= 0.033) did not change significantly. Prevalence of non-HDL cholesterol ≥ 160 mg/dl (r^2 ^= 0.089), remnant cholesterol ≥ 25 mg/dl (r^2 ^= 0.155), and total: HDL cholesterol ≥ 4.0 (r^2 ^= 0.120) and ≥ 5.0 (r^2 ^= 0.112) increased.

Prevalence of dyslipidemias categorized according to the ATP-3 report [25] is shown in Table 3. In all cohorts the prevalence of various forms of lipid abnormalities increased with age. There is a sharp increase in dyslipidemia prevalence from JHW-1 to JHW-2 study. Curve-estimation regression analysis shows significant increase in non-HDL cholesterol, remnant cholesterol, triglycerides and total:HDL cholesterol. Graphical trends in age-adjusted prevalence (%) of dyslipidemias in JHW-1, JHW-2, JHW-3 and JHW-4 studies respectively show (Figure 3) insignificant changes in high total cholesterol (23.7, 35.1, 27.1, 26.1, least squares regression r^2 ^0.02), high LDL cholesterol ≥ 130 mg/dl (21.9, 36.2, 31.1, 22.1, r^2 ^0.13), and low HDL cholesterol < 40 mg/dl (42.9, 53.3, 55.4, 33.7, r^2 ^0.02). Significant increase is observed in prevalence of high non-HDL cholesterol (20.8, 33.5, 27.4, 26.6, r^2 ^0.20), high remnant cholesterol (40.1, 40.3, 30.1, 60.6, r^2 ^0.15), high total:HDL cholesterol ratio (≥ 5.0: 20.1, 47.6, 53.2, 26.4, r^2 ^0.15 and ≥ 4.0: 60.5, 74.4, 77.7, 66.6, r^2 ^0.12) and high triglycerides (22.8, 28.2, 17.5, 34.2, r^2 ^0.13). Secular trends in men (data not shown) reveal significant increase in prevalence of high non-HDL cholesterol (r^2^0.22), high remnant cholesterol (r^2 ^0.34), high total:HDL cholesterol and ≥ 4.0 (r^2 ^0.28), and high triglycerides (r^2 ^0.31) while in women prevalence of high non-HDL cholesterol (r^2 ^0.11) increased and that of low HDL cholesterol < 40 mg/dl decreased (r^2^0.38).

**Table 3:** Age-specific prevalence rates (%) and trends in dyslipidaemia in various studies

Lipid Parameters	JHW-1 (n = 276)	JHW-2 (n = 926)	JHW-3 (n = 374)	JHW-4 (n = 500)	Linear curve-estimation regression coefficient. Multiple R (p value)
Cholesterol ≥200 mg/dl					
20–29	12 (17.6)	46 (24.6)	11 (13.4)	16 (17.0)	0.034 (0.088)
30–39	30 (29.1)	107 (36.9)	29 (27.1)	36 (28.8)	
40–49	24 (34.3)	103 (43.3)	41 (38.3)	41 (27.5)	
50–59	12 (34.3)	107 (50.7)	31 (39.7)	60 (45.5)	
Age adjusted, 95% CI	26.3 (20.1–32.5)	35.1 (32.0–38.2)	25.6 (21.1–30.0)	26.0 (22.1–29.8)	
LDL cholesterol ≥130 mg/dl					
20–29	9 (13.2)	50 (26.7)	14 (17.1)	15 (16.0)	0.062 (0.002)
30–39	35 (34.0)	111 (38.3)	40 (37.4)	32 (25.6)	
40–49	21 (30.0)	101 (42.4)	42 (39.3)	28 (18.8)	
50–59	9 (25.7)	108 (51.2)	36 (46.2)	52 (39.4)	
Age adjusted, 95% C	24.2 (18.1–30.2)	36.2 (33.1–39.3)	31.0 (26.3–35.7)	22.2 (18.6–25.8)	
HDL cholesterol < 40 mg/dl					
20–29	36 (52.9)	90 (48.1)	48 (58.5)	23 (24.5)	0.136 (< 0.001)
30–39	42 (40.8)	166 (57.2)	57 (53.3)	51 (40.8)	
40–49	29 (41.4)	144 (60.5)	58 (54.2)	58 (38.9)	
50–59	16 (45.7)	103 (48.8)	41 (52.6)	49 (37.1)	
Age adjusted, 95% CI	46.2 (40.3–52.1)	53.3 (50.1–56.6)	55.4 (52.2–58.6)	33.7 (29.6–37.8)	
Non-HDL cholesterol ≥160 mg/dl					
20–29	10 (14.7)	41 (21.9)	11 (13.4)	19 (20.2)	0.026 (0.196)
30–39	31 (30.1)	106 (36.5)	32 (29.9)	36 (28.8)	
40–49	20 (28.6)	100 (42.0)	43 (40.2)	41 (27.5)	
50–59	8 (22.9)	104 (49.3)	35 (44.9)	53 (40.2)	
Age adjusted, 95% CI	23.0 (18.0–28.0)	33.5 (30.5–36.5)	27.4 (22.9–31.9)	26.6 (22.7–30.5)	
Total/HDL ratio ≥5.0					
20–29	12 (17.6)	67 (35.8)	31 (37.8)	16 (17.0)	0.031 (0.123)
30–39	23 (22.3)	155 (53.4)	62 (57.9)	39 (31.2)	
40–49	19 (27.1)	135 (56.7)	72 (67.3)	37 (24.8)	
50–59	10 (28.6)	118 (55.9	53 (67.9)	62 (47.0)	
Age adjusted, 95% CI	22.2 (17.3–27.1)	47.6 (44.4–50.8)	53.2 (48.1–58.3)	26.3 (22.4–30.2)	
Total/HDL ratio ≥4.0					
20–29	41 (60.3)	119 (63.6)	46 (56.1)	51 (54.3)	0.006 (0.756)
30–39	64 (62.1)	221 (76.2)	82 (76.6)	83 (66.4)	
40–49	33 (47.1)	191 (80.3)	86 (80.4)	90 (60.4)	
50–59	21 (63.6)	166 (78.7)	64 (82.1)	104 (78.8)	
Age adjusted, 95% CI	58.6 (52.8–64.4)	72.5 (69.6–75.4)	70.1 (65.5–74.7)	62.0 (57.8–66.3)	
Triglycerides ≥150 mg/dl					
20–29	11 (16.2)	38 (20.3)	11 (13.4)	24 (25.5)	0.047 (0.019)
30–39	24 (23.3)	97 (33.4)	17 (15.9)	49 (39.2)	
40–49	26 (37.1)	71 (29.8)	23 (21.5)	60 (40.3)	
50–59	15 (42.9)	80 (37.9)	22 (28.2)	52 (39.4)	
Age adjusted, 95% CI	25.7 (19.5–31.8)	28.2 (25.3–31.1)	17.5 (13.7–21.4)	34.2 (30.0–38.3)	
Remnant cholesterol ≥25 mg/d					
20–29	20 (29.4)	55 (29.4)	18 (22.0)	48 (51.1)	0.143 (< 0.001)
30–39	38 (36.9)	135 (46.6)	30 (28.0)	85 (68.01)	
40–49	40 (57.1)	105 (44.1)	41 (38.3)	97 (65.1)	
50–59	19 (54.3)	112 (53.1)	37 (47.4)	87 (65.9)	
Age adjusted, 95% CI	40.1 (34.3–45.9)	40.3 (37.1–43.4)	30.1 (25.4–34.7)	60.6 (56.4–64.9)	

We correlated changing lipid levels with socioeconomic status and generalized and truncal obesity. Educational level has been used as marker of socioeconomic status [23] and the age-adjusted prevalence of literacy greater than primary education (> 5 years of formal education) in successive studies increased significantly in men (66.4, 83.0, 98.4, 90.7, r^2 ^0.69) as well as women (30.3, 67.8, 99.1, 87.5, r^2 ^0.75). Significantly increasing trends in overweight or obesity (r^2 ^men 0.84, women 0.78) as well as truncal obesity (r^2 ^men 0.28, women 0.20) in different JHW cohorts were also observed [24]. Cross correlation analysis using two-line regression revealed that increasing educational status correlated significantly with obesity (r^2 ^men 0.98, women 0.99) as well as truncal obesity (r^2 ^men 0.71, women 0.90). For various lipoprotein lipids a two-line regression analysis reveals more significant relationship of truncal obesity as compared to generalized obesity with various lipid parameters (Table 4). Increasing truncal obesity correlates significantly with high total cholesterol (r^2 ^0.77, p = 0.011), LDL cholesterol (r^2 ^0.74, p = 0.014), non-HDL cholesterol (r^2 ^0.88, p = 0.003), and total:HDL cholesterol ≥ 5.0 (r^2 ^0.59, p = 0.037) and ≥ 4.0 (r^2 ^0.77, p = 0.011). Obesity correlates weakly with increasing LDL cholesterol (r^2 ^0.46, p = 0.069), non-HDL cholesterol (r^2 ^0.42, p = 0.082), total:HDL cholesterol ratio ≥ 5 (r^2 ^0.46, p = 0.071) and total:HDL cholesterol ratio ≥ 4 (r^2 ^0.48, p = 0.061) (Table 4).

**Table 4:** Two-line regression analysis (r^2^, and p value) of association of high educational status (> primary education), obesity (BMI ≥ 25 kg/m^2^) and truncal obesity (waist:hip > 0.9) with various lipid parameters.

**Lipid variable**	**Educational status**	**Obesity**	**Truncal obesity**
Total Cholesterol ≥ 200 mg/dl	0.450 (0.072)	0.322 (0.120)	0.773 (0.011)
LDL Cholesterol ≥ 130 mg/dl	0.529 (0.051)	0.462 (0.069)	0.737 (0.014)
HDL Cholesterol < 40 mg/dl	0.347 (0.109)	0.352 (0.107)	0.477 (0.064)
Non-HDL Cholesterol ≥ 160 mg/dl	0.531 (0.050)	0.421 (0.082)	0.884 (0.003)
Remnant cholesterol ≥ 25 mg/dl	-0.211 (0.179)	-0.257 (0.152)	-0.0711 (0.305)
Total:HDL cholesterol ratio ≥ 5.0	0.469 (0.067)	0.456 (0.071)	0.593 (0.037)
Total:HDL cholesterol ratio ≥ 4.0	0.535 (0.049)	0.480 (0.061)	0.768 (0.011)
Triglycerides ≥ 150 mg/dl	-0.111 (0.259)	-0.196 (0.189)	0.099 (0.272)

## Discussion

This study shows that there is a high prevalence of various forms of lipoprotein abnormalities in Indian urban subjects. Secular trends reveal increasing mean levels of total-, LDL-, non HDL-, and remnant cholesterol, total:HDL cholesterol ratio and triglycerides and decline in HDL cholesterol. Prevalence of high non-HDL cholesterol, remnant cholesterol, and total-HDL cholesterol ratio increased. These changes correlate significantly with increasing education (socioeconomic status) and truncal obesity. Most of the lipid abnormalities are markers of dietary excess, low physical activity and increasing obesity [27]. The present study confirms that increasing obesity manifest as truncal obesity, due to population-wide sedentary lifestyle and high calorie intake [28,29], leads to increase in multiple dyslipidemias. We have previously reported increase in prevalence of coronary heart disease [2] in urban Indian populations and the present study suggests that increasing non-HDL cholesterol, cholesterol remnants, and total-HDL cholesterol ratio are important risk factors. The importance of these dyslipidemias has been highlighted in multiple prospective studies from other countries [30-32].

Rise and fall of cholesterol and other lipoproteins associated with changing cardiovascular mortality and coronary heart disease incidence has been well documented in many developed countries [7,8,25]. There is paucity of similar data from developing countries. Data of the present study has significant healthcare policy and pharmacoeconomic implications because more than 40% of the world's population is in India and China. A economies of these countries boom [33] and individual buying capacity increases the lifestyle changes shall lead to massive increase in lipid levels fuelling cardiovascular epidemic as observed in the present study in an Indian urban population.

In China two large scale surveys have been carried out to determine prevalence of lipid abnormalities [34,11]. The first survey in 1992 reported greater lipid values in urban as compared to rural populations [34]. Among 9477 subjects the mean ± SD cholesterol at urban sites in China was 181.6 ± 32 to 184.8 ± 38 mg/dl in men and 187.5 ± 33 to 187.6 ± 42 in women. The values were 15–25 mg lower in rural subjects [34]. Prevalence of hypercholesterolemia ≥ 200 mg/dl was 29.1–31.0% in urban subjects and 7.7–20.0% rural subjects. Second survey in 2004 was a population based epidemiological study among 15540 adults and reported mean ± SEM cholesterol of 193.0 ± 0.7 in urban men and 196.4 ± 0.7 in urban women [11]. These levels were significantly greater than the 1992 study. This study also reported lower urban-rural gap in cholesterol levels (10–11 mg/dl more in the urban). Age-adjusted prevalence of hypercholesterolemia was 39.8% in urban men, 44.1% in urban women, 30.2% in rural men and 31.7% in rural women. Age-adjusted prevalence rates for hypercholesterolemia are lower in our study (Table 3). Prevalence of low HDL cholesterol < 40 mg/dl in Chinese urban subjects were 29.5% in men and 14.6% in women aged 35–74 years which is lower than reported in our subjects. These studies did not report prevalence of hypertriglyceridemia or high total:HDL cholesterol ratios. Another study from China reported changing trends of cardiovascular risk factors in different socioeconomic groups but did not comment on lipid levels [35]. The Korea National Health and Nutrition Examination Survey (2001) in subjects 20–79 years reported mean cholesterol of 187.8 ± 33 mg/dl in men and 185.3 ± 34 mg/dl in women, HDL cholesterol of 44.1 ± 10 mg/dl in men and 48.5 ± 10 mg/dl in women, and triglycerides of 155.1 ± 83 in men and 118.8 ± 68 mg/dl in women [12]. Prevalence of hypercholesterolemia ≥ 240 mg/dl was in 7.1% men and women while low HDL cholesterol levels < 35 mg/dl were in 35.1% men and 17.8% women. These results are similar to studies from China and prevalence of low HDL cholesterol is less than our studies. In a study in 417 Mexican cities among 2256 of 15607 enrolled subjects, 20–69 years, mean cholesterol was 182.7 mg/dl, triglycerides 213.4 mg/dl, HDL cholesterol 38.3 mg/dl and LDL cholesterol 116.4 mg/dl [36]. Prevalence of hypercholesterolemia was in 27.1%, low HDL, defined by ATP-3 guidelines, in 48.4% and hypertriglyceridemia in 42.3% in men and women. These values are similar to our studies. We have used cut-off for diagnosis of low HDL cholesterol in consonance with the Asian studies to make the data regionally comparable. The ATP-3 guidelines [25] suggest different HDL cholesterol thresholds for men (< 40 mg/l) and women (< 50 mg/dl). We analysed data based on cut-off of < 40 mg/dl because when the cut-off of < 50 mg/dl was used more than 90% of study subjects fell in dyslipidemia category. On the other hand in Sub-Saharan African countries very low mean population total cholesterol levels, 140–160 mg/dl, and very high HDL cholesterol levels, 50–65 mg/dl have been reported [37]. Dietary and other lifestyle habits could be a factor as in traditional hunter-gatherer populations similar findings have been reported [38].

Secular trends in cholesterol lipoproteins in developed countries reveal a decline in mean serum total cholesterol and LDL cholesterol levels. In USA, periodical cross sectional surveys from 1960–2002 have reported that mean ± SEM total cholesterol in adults 20–74 years decreased from 222 ± 1.5 mg/dl in 1960–62, to 216 ± 0.8 in 1971–74, 215 ± 1.1 in 1976–80, 204 ± 0.7 in 1988–1994 ad 203 ± 0.9 mg/dl in 1999–2002 [39]. Values of other lipids were reported in latter studies and as compared from years 1976–80 to 1999–2002 levels of LDL cholesterol declined from 138 mg/dl to 123 mg/dl, HDL cholesterol levels increased from 49.7 mg/dl to 51.0 mg/dl and triglyceride levels increased from 114 mg/dl to 122 mg/dl. Age-adjusted prevalence of hypercholesterolemia ≥ 240 mg/dl decreased from 20 ± 0.6% during 1988–1994 to 17 ± 0.6% during 1999–2002. Similar declines in population cholesterol levels and prevalence of hypercholesterolemia has been reported from North American and Western European cohorts of Seven Countries Study [7]. The decline in total and LDL cholesterol has been attributed to documented decreases in dietary intake of saturated fats and cholesterol [40]. However, recent evidence suggests that the decline in USA may have been due to increased use of medications rather than positive lifestyle changes [40]. It is also suggested that the slower decline in recent years is likely due to increase in obesity among adults and the observed increase in triglyceride levels is a marker [39]. In the present study too, it is observed that increasing obesity is important determinant of increases in total, non-HDL, and LDL cholesterol and triglycerides. This augurs more adverse lipid profiles worldwide unless the obesity epidemic is controlled.

This study has multiple limitations as well as strengths. The variable and low response rates in some cohorts make the data tenuous but the age-structure of the studied cohorts was similar to the local populations and therefore the data can be generalized for evaluation of risk factor trends. Small number of subjects in each of the studies and age-specific subgroup could also be concern but the sample sizes have been determined using available recommendations for the prevalence of cardiovascular risk factors in a community [41] and are considered appropriate for inter-group comparisons. We have determined both age-adjusted mean levels of various lipoproteins as these could be earliest population-level change and these show significant trends. Prevalence rates are robust evidence of population level change and the present study using simple meta-regression techniques shows significant trends in important lipid abnormalities. This type of meta-regression is used for combining clinical trials as well as epidemiological studies using pre-defined end points [42]. Generalizability of the study results to the local urban population or to the whole country may not be appropriate at this time as socioeconomic structure of the country is so different from locality to locality and town to town [43]. A major strength of the study is use of similar assessment methodologies that make the observations comparable. Another strength is determination of different types of lipoprotein abnormalities and cholesterol ratios which have emerged as important risk factor. The study definitively shows that biological risk factors (lipids) are causally related to increasing obesity and to increasing socioeconomic status as measured by educational status. It has been previously reported that up to a certain level of socioeconomic status (gross national product) the risk factors tend to increase and once a particular per capita income is achieved the risk factors tend to decline with increasing socioeconomic status [44]. Indeed, a more careful assessment of the trends of dyslipidemias (Figure 3) reveals that risk factors in educated Indian middle-class subjects may be starting to level-off as observed in JHW-3 and JHW-4 studies. This indicates importance of evolving socioeconomic changes as an important driver as well as controller of cardiovascular diseases [44].

Low HDL cholesterol and high total:HDL cholesterol are important cardiovascular risk factors. Multiple prospective studies have identified the importance of low HDL cholesterol as cardiovascular risk factor [25]. Importance of total:HDL ratio has been highlighted in the Physicians Health Study that reported relative risk (RR) of acute myocardial infarction in the top vs. bottom quintile of total:HDL cholesterol ratio was 3.73 (95% confidence interval 1.95–7.12) and was substantially greater than total cholesterol (RR 1.86; 1.05–3.28), HDL cholesterol (0.38; 0.21–0.69), and apolipoprotein B (2.50; 1.31–4.75) [45]. The INTERHEART Study also reported that ratio of apolipoprotein B/A_1 _was the most important risk factor for acute myocardial infarction in South Asians [46]. It has also been reported that in patients receiving statin therapy levels of non-HDL cholesterol, apolipoprotein B, and total:HDL cholesterol ratio of ≥ 4.0 were more important than other lipid parameters [47]. Increasing ratio in this Indian urban population along with increasing non-HDL cholesterol and falling HDL cholesterol levels associated with increasing socioeconomic status and obesity shows the appropriate direction for prevention effort. Increasing socioeconomic status of Indians has to be complemented with intensive public health education and policy changes at the national level [2,48] for cardiovascular disease prevention.

In conclusion, this report is first in a low income country – India- that demonstrates cross-sectional and longitudinal trends in dyslipidemia and its causal relationship with adiposity and socioeconomic status. Data analysis of such a cohort reveals non-similarities (increasing non-HDL cholesterol, triglycerides, and total-HDL ratio) as well as similarities (increasing socioeconomic status and obesity) vis-à-vis other developing and developed regions of the world [49]. The inferences that can be translated for implications to health care policies and practice, medical education and research is beyond the scope of this publication.

## Competing interests

SG is a full time employee of Merck, USA in Singapore. This company has significant interest in lipid modifying medicines. Other authors have no conflict of interests relevant to this article.

## Authors' contributions

RG conceived, designed, overviewed data collection and implemented the study, provided critical academic inputs and wrote the first draft and subsequent revisions of the manuscript; SG provided academic inputs, contributed to the first draft of the article and revisions; AA helped in data collection and analyses; VK overlooked biochemical measurements, helped in data collection and analysis; KG helped in data analyses; VPG jointly conceived, designed and overviewed data collection, provided statistical inputs and participated in writing of the article. All authors have read and approved the final manuscript.
